# Randomisierte kontrollierte multizentrische Studie zur Albuminersatztherapie im septischen Schock (ARISS)

**DOI:** 10.1007/s00101-021-00952-5

**Published:** 2021-03-31

**Authors:** Yasser Sakr, Luciano Gattinoni, SepNet-Critical-Care-Trials-Gruppe, C. Schulze, J. G. Westphal, R. Pfeifer, M. Frizenwanger, F. Fichtner, P Simon, S Rasche, S Schering, S. Petros, L. Weidhase, B. Pasieka, P. Appelt, K. Wartenberg, D. Michalski, A. Nierhaus, D. Jarczak, A. Zarbock, M. N. Weiler, I. Lautenschläger, G. Elke, T. Becher, M. Kott, F. Roghmann, M. Senkal, U. Hermann Frey, P. Bischof, U. Jaschinski, P. Deetjen, O. Mörer, M. Quintel, L. Gattinoni, J. Briegel, M. Rehm, B. Kapfer, J. Martin, S. Utzolino, L. Kousoulas, C. Scheer, S. O. Kuhn, J. Lehmke, J. Ebbinghaus, I. Tanev, A. Schmeisser, M. Holubek, J. Handerer, D. Jacob, U. Lodes, A. Weyland, U. Günther, C. Putensen, C. Weissbrich, J. C. Schewe, S. Ehrentraut, F. Fiedler, P. Tepaß

**Affiliations:** 1 Klinik für Anästhesiologie und Intensivtherapie, Universitätsklinikum Jena, Am Klinikum 1, 07743 Jena, Deutschland; 2 Klinik für Anästhesiologie, Rettungs- und Intensivmedizin, Universitätsmedizin Göttingen, Göttingen, Deutschland

## Hintergrund & Hypothesen

Neben der onkotischen Funktion verfügt Albumin über vielfältige weitere Eigenschaften, darunter Bindung und Transport verschiedener endogener Moleküle [1, 2], eine antiinflammatorische [3] und antioxidative Wirkung [4] und die Modulation des Stickstoffmonoxidmetabolismus [4]. In einer randomisierten Studie bei 7000 kritisch kranken Patienten wurde der mögliche Einfluss einer Volumenersatztherapie mit Humanalbumin, 4 %, im Vergleich zur Volumenersatztherapie nur mit Kristalloiden auf das Outcome untersucht [5]. Obwohl die Überlebensraten zwischen den beiden Gruppen ähnlich waren, zeigte eine Post-hoc-Analyse an 1218 Patienten mit schwerer Sepsis eine erniedrigte Mortalität in der Albumingruppe im Vergleich zur Kontrollgruppe [6]. Folglich untersuchte die randomisierte kontrollierte ALBIOS-Studie (*Alb*umin *I*talian *O*utcome *S*epsis study) den möglichen Einfluss einer Albumingabe und Aufrechterhaltung des Albumin-Serum-Wertes bei 1810 Patienten mit schwerer Sepsis und septischem Schock auf das Outcome [7]. Die Studie zeigte keinen Outcome-Unterschied zwischen den Studiengruppen; zwei interessante Signale waren jedoch ersichtlich: Erstens wurde eine Tendenz für einen potenziellen Überlebensvorteil einer Albumintherapie bei Patienten, bei denen die Therapie 6–24 h nach Eintritt der Sepsis begonnen wurde, festgestellt. Zweitens zeigte sich bei 1121 Patienten mit septischem Schock eine erniedrigte 90-Tage-Mortalität in der Albumingruppe im Vergleich zu Patienten der Kontrollgruppe. Zusammengenommen weist die aktuelle Datenlage darauf hin, dass die Albumingabe bei schwerer und fortgeschrittener Sepsis mit Beeinträchtigung des protektiven Effekts des Serumalbumins einen Überlebensvorteil bringen könnte. Es gibt bislang keine randomisierte Studie, die diese Hypothese bei Patienten mit septischem Schock ausreichend untersuchte.

Das Ziel der klinischen Prüfung ARISS (engl. für: „albumin replacement in septic shock“) ist es, den Einfluss einer Albumingabe und die Aufrechterhaltung eines Albumin-Serum-Werts von mindestens 30 g/l für 28 Tage nach Eintritt des septischen Schocks im Vergleich zur Volumenersatztherapie ohne Albumin auf das Überleben der Patienten zu untersuchen. Die Studienhypothese ist, dass eine Albumingabe, begonnen innerhalb von 6–24 h nach dem Eintritt des septischen Schocks, und die Aufrechterhaltung eines Albumin-Serum-Werts von mindestens 30 g/l für 28 Tage nach Eintritt des septischen Schocks im Vergleich zur Volumenersatztherapie ohne Albumin die 90-Tage-Gesamtmortalität verringern werden.

## Details der Studie

### Studiendesign

Die ARISS ist eine prospektive, multizentrische, randomisierte, kontrollierte, parallel-gruppierte, unverblindete, interventionelle klinische Prüfung (Phase IIIb) nach dem Arzneimittelgesetz.

### Einschlusskriterien

- Vorliegen eines septischen Schocks [8, 9],
- Beginn des septischen Schocks vor weniger als 24 h,
- Alter: ≥ 18 Jahre,
- potenziell gebärfähige Patientin: negativer Schwangerschaftstest.

### Ausschlusskriterien

- Infauste Prognose,
- „End-of-life“-Entscheidungen vor Einwilligung bekannt,
- vorherige Teilnahme an dieser Studie oder Teilnahme an einer anderen interventionellen klinischen Prüfung innerhalb der zurückliegenden 3 Monate,
- Schockzustände, die durch andere Ursachen erklärbar sind,
- anamnestisch bekannte Überempfindlichkeit gegenüber Albumin oder einem sonstigen Bestandteil des Prüfpräparates,
- Krankheitszustand, der gegen eine Anwendung von Albumin spricht,
- Umstand, bei dem Albumingabe vorteilhaft ist,
- Stillzeit.

### Primärer Endpunkt

Als primärer Endpunkt wird die 90-Tage-Gesamtmortalität gewählt.

### Sekundäre Endpunkte

- 28- bzw. 60-Tage-Mortalität,
- Mortalitätsraten auf der Intensivstation und während des Krankenhausaufenthaltes,
- Organdysfunktion/-versagen nach Sequential Organ Failure Assessement (SOFA)-Score,
- Intensivstation- bzw. Krankenhausverweildauer,
- Tage ohne Beatmungspflicht, Tage ohne Vasopressoren,
- Kosten-Nutzen-Parameter der Volumenersatztherapie,
- Gesamtflüssigkeitsaufnahme und Gesamtflüssigkeitsbilanz,
- Auftreten von „adverse events“ (AE) und „severe adverse events“ (SAE).

## Interventionen

Bei dem Prüfpräparat der klinischen Prüfung handelt es sich entweder um Albutein® 200 g/l oder Plasbumin® 20, beide zugelassene Arzneimittel mit dem Wirkstoff humanes Albumin, 200 g/l. In der Interventionsgruppe (Albumingruppe) muss die Gabe der Startdosis des Prüfpräparates (60 g Humanalbumin über 2–3 h) innerhalb von 6–24 h nach Beginn des septischen Schocks begonnen werden. Die Gabe des Prüfpräparates erfolgt je nach tagesaktuellem Albumin-Serum-Wert. Die Dosisanpassung findet nach einem vorgegebenen Schema statt, mit dem Ziel, einen Albumin-Serum-Wert von mehr als 30 g/l aufrechtzuerhalten für maximal 28 Studientage nach der Randomisierung und nur, solange der Teilnehmer auf der Intensivstation behandelt wird.

## Mitwirkung

### Voraussetzungen

Alle Prüfzentren werden anhand eines Selektionsbesuchs auf ihre Eignung als Prüfzentrum überprüft. Zenten in Deutschland sind eingeladen, an der Studie teilzunehmen.

### Ansprechpartner für Interessenten

apl-Prof. Dr. Yasser Sakr: Klinik für Anästhesiologie und Intensivtherapie, Uniklinikum Jena, Am Klinikum 1, 07743 Jena, E‑Mail: yasser.sakr@med.uni-jena.de.

## Statistik

### Fallzahlplanung

Es wird angenommen, dass die 90-Tage-Mortalität im Kontrollarm etwa 50 % beträgt. Für die Fallzahlschätzung wird eine 15 %ige Reduktion der 90-Tage-Sterblichkeit angenommen, d. h. eine absolute Reduktion um 7,5 Prozentpunkte auf 42,5 % (relatives Risiko 1,19). Ein Mantel-Haenszel-Chi^2^-Test auf einem zweiseitigen Signifikanzniveau von 0,05 mit einer Power von 80 % benötigt 1412 auswertbare Patienten, 706 pro Arm, um einen solchen Effekt nachzuweisen. Unter Annahme der Drop-out-Rate von 15 % müssen 1662 Patienten randomisiert werden.

### Randomisierung

Die Randomisierung erfolgt stratifiziert nach Serumlactat innerhalb der 24 h vor Einschluss ≤ 8 mmol/l vs. > 8 mmol/l und Zentrum in die Behandlungsgruppen; Albumingruppe und Kontrollgruppe ohne Albumingabe.

### Subgruppenanalysen

Der primäre Endpunkt und ausgewählte sekundäre Endpunkte werden deskriptiv für folgende Subgruppen dargestellt: Baseline-Lactat ≤ 8 mmol/l vs. > 8 mmol/l, „SOFA total und subscores“, Simplified Acute Physiology (SAPS II-Score), Acute Physiology and Chronic Health Evaluation (APACHE II-Score): nach Baseline-Quartilen.

## Ethik

### Ethische Aspekte

Die klinische Prüfung wurde von der zuständigen Ethikkommission zustimmend bewertet (Ethikkomission, Uniklinikum Jena, Bachstraße 18, 07740 Jena, Germany; Reg.-Nr.: 2018-1227-AMG_ff. Protokoll-Nr.: ZKSJ0122_ARISS). Die Ethikkommissionen der beteiligten Prüfzentren sollen die klinische Prüfung vor dem Beginn der Patientenrekrutierung in den jeweiligen Zentren zustimmend bewerten.

Die Patienteninformation erfolgt mündlich und schriftlich mit den vorgegebenen Formularen. Die Einwilligungserklärung zur Teilnahme an der klinischen Prüfung muss von der einwilligenden Person eigenhändig datiert und unterzeichnet werden.

Die Registrierung der klinischen Prüfung erfolgte vor Randomisierung des ersten Patienten in Clinicaltrials.gov (NCT03869385) und in dem von der WHO anerkannten Deutschen Register Klinischer Studien (DRKS; www.drks.de).

### Monitoring

Das Monitoring wird nach den Standardarbeitsanweisungen des Zentrum für klinische Studien (ZKS) Jena durchgeführt.

### Komitee für Sicherheit 

Das Expertengremium erhält in regelmäßigen Abständen oder bei gegebenem Anlass alle sicherheitsrelevanten Daten und Informationen, um eine unabhängige Bewertung der Sicherheit der klinischen Prüfung durchzuführen.

## Meilensteine

### Studiendauer

Für die Rekrutierung der Patienten sind ca. 36 Monate vorgesehen.

### Ablauf der klinischen Prüfung

Das erste Zentrum wurde am 16.09.2019 initiiert. Initiierungsbesuche wurden erfolgreich in insgesamt 26 Zentren durchgeführt. Es wurden insgesamt 230 Patienten bis zum 01.03.2021 randomisiert. Der Prüfplan wurde in englischer Sprache veröffentlicht [10].

## Studiengruppe/Expertise

Die SepNet-Studiengruppe vereint Experten verschiedener Fachdisziplinen, die auf dem Gebiet der klinischen und experimentellen Sepsisforschung tätig sind. Sie haben in diesem Bereich beträchtliche Erfahrung und Interesse an klinischen Studien mit Sepsispatienten. Die organisatorische Struktur der klinischen Prüfung ist in Tabelle S1 (Zusatzmaterial online) dargestellt.

## Supplementary Information


