# Supplementary material for: Randomized controlled multicenter study of albumin replacement therapy in septic shock (ARISS)
Source: Anaesthesist. 2021 Mar 31;70(6):528–30. [Article in German] doi: 10.1007/s00101-021-00952-5 (PMC8189957; doi:10.1007/s00101-021-00952-5)
Supplement: Supplementary file 1 [file 101_2021_952_MOESM1_ESM.pdf]

Tabelle Organisatorische Struktur der klinischen Prüfung

Zusatzmaterial zum Beitrag „Randomisierte kontrollierte multizentrische Studie zur Albuminersatz-therapie im septischen Schock - ARISS“ von Sakr Y, Gattinoni L, SepNet-Critical-Care-Trials-Gruppe (2021) in *Der Anaesthesist.*

Beitrag und Zusatzmaterial stehen Ihnen auf www.springermedizin.de zur Verfügung. Bitte geben Sie dort den Beitragstitel in die Suche ein.

**Funktion/ Qualifikation**	**Name**	**Affiliation**
Sponsor gemäß Arzneimittelgesetz	Friedrich Schiller University Jena	
Sponsorbevollmächtigter und Leiter der klinischen Prüfung (LKP)	Prof. Dr. Yasser Sakr	Universitätsklinikum Jena, Klinik für Anästhesiologie und Intensivmedizin
Vertreter des LKP	Prof. Dr. Michael Bauer	Universitätsklinikum Jena, Klinik für Anästhesiologie und Intensivmedizin
Protokollkomitee	Prof. Dr. Yasser Sakr,<br>Prof. Dr. Michael Bauer,<br><br>Dr. Ulrike Schumacher<br>Dr. Maria Breternitz<br>Dr. Sabine Barta<br><br>Prof. Dr. Michael Hartmann,<br>PD Dr. Michael Kiehntopf<br><br>Prof. Dr. Luciano Gattinoni<br>Prof. Dr. Michael Quintel	Universitätsklinikum Jena, Klinik für Anästhesiologie und Intensivmedizin<br><br>Universitätsklinikum Jena, Zentrum für Klinische Studien (ZKS)<br><br>Universitätsklinikum Jena<br><br>Universitätsklinikum Göttingen
Biometriker	Dr. Ulrike Schumacher	Universitätsklinikum Jena, Zentrum für Klinische Studien (ZKS)

**Funktion/ Qualifikation**	**Name**	**Affiliation**
Projektmanagement	Dr. Franziska Weber	Universitätsklinikum Jena, Zentrum für Klinische Studien
Datenmanagement	Aicko Helbig	Universitätsklinikum Jena, Zentrum für Klinische Studien
Monitoring	Dr. Christine Gampe<br><br>Silvia Apel	Universitätsklinikum Jena, Zentrum für Klinische Studien<br>Universitätsklinikum Göttingen, Zentrum für Klinische Studien
Pharmacovigilanz (Safety-Management)	Dr. Mariann Städtler<br>Sandra Birr	Universitätsklinikum Jena, Zentrum für Klinische Studien
zentrale Apotheke für Lagerung und Versand des Prüfpräparates an Apotheken der Prüfzentren	Prof. Dr. Michael Hartmann	Apotheke, Universitätsklinikum Jena
Apotheken fürBereitstellung des Prüfpräparates an die Prüfzentren	local pharmacy of the respective trial centre	
Ökonomische Analyse	Prof. Dr. Michael Hartmann	Apotheke, Universitätsklinikum, Jena
Komitee für Sicherheit –SMC	Prof. Dr. Ricard Ferrer<br><br><br>Prof. Dr. Marco Ranieri<br><br><br>Dr. Hassane Njimi	Dept. of Intensive Care, Hospital Universitari Vall d 'Hebron Barcelona, Spain<br>Dept. of Anesthesiology and Intensive Care, Sapienza University of Rome, Italy<br>Dept. of Critical Care, Erasme Hospital, Free University of Brussels, Belgium
zuständige Bundesoberbehörde (BOB)	Paul-Ehrlich-Institut (PEI), Bundesinstitut für Impfstoffe und biomedizinische Arzneimittel	
federführende Ethik-Kommission	Ethik-Kommission der Friedrich-Schiller-Universität Jena	

Funktion/ Qualifikation	Name	Affiliation
Prüfzentren	SepNet - Critical Care Trials Group, Germany	
